# *In Vitro Fertilization* and Placenta Accreta (spectrum): A Systematic Review

**DOI:** 10.5935/1518-0557.20250192

**Published:** 2026

**Authors:** Ana Carolina Wickert Theisen, Nathália Fritsch Camargo, Talita Colombo

**Affiliations:** 1 UFCSPA, Porto Alegre, Rio Grande do Sul, Brazil; 2 PUCRS, Porto Alegre, Rio Grande do Sul, Brazil

**Keywords:** placenta accreta, fertilization *in vitro*, assisted reproductive techniques, embryo transfer

## Abstract

*In vitro* fertilization (IVF) has transformed infertility treatment, providing options for individuals struggling to conceive naturally, with significant success rates, particularly in women under 35. However, IVF pregnancies are associated with potential obstetric complications, notably placenta accreta spectrum (PAS), which necessitates early diagnosis and a multidisciplinary approach to safeguard maternal-fetal health. This systematic review involved a comprehensive search of databases such as PubMed, Cochrane Library, BVS, Embase, Web of Science, and Scopus using specific terms related to IVF and PAS. Original studies comparing spontaneous pregnancies to IVF pregnancies were included, while case reports and non-peer-reviewed articles were excluded. Data extraction was standardized, and study quality was assessed using the Newcastle-Ottawa tool. The analysis covered thirteen articles involving 252,295 assisted reproductive technology pregnancies and over 16 million spontaneous pregnancies. A key finding is the increased risk of PAS in IVF pregnancies, with some studies indicating that frozen embryo transfer (FET) poses a higher risk than fresh embryo transfer. Additional complications include hypertension, preeclampsia, and intrauterine growth restriction (IUGR). Interestingly, some research suggests a lower Intensive Care Unit admission rate for women with placenta accreta after IVF compared to those with spontaneous conception. This indicates that while assisted reproductive technology is an independent risk factor for PAS, it presents a unique risk profile. The review emphasizes the need for further research into maternal outcomes related to PAS after assisted reproductive techniques (ART) and highlights the importance of specialized follow-up and rigorous prenatal diagnosis to minimize complications in these pregnancies.

## INTRODUCTION

*In vitro* fertilization (IVF) has revolutionized the treatment of infertility, offering hope to individuals facing complex reproductive challenges. Since its introduction into clinical practice in the late 1970s (Fishel, 2018), IVF has significantly evolved in terms of efficacy and safety, becoming a widely used technique in cases of endometriosis, ovulatory disorders, tubal-peritoneal factors, and male infertility factors. Between 2020 and 2024, in Brazil 544,983 embryos have been vitrified, across 216 assisted reproduction centers, with 68% of these procedures performed in the southeastern region. Of these, 65,627 were transferred, resulting in a clinical pregnancy rate of approximately 27.4% in patients under 35 years old and 18.5% in those aged 35 or older (ANVISA - Agência Nacional de Vigilância Sanitária, 2025). In parallel with the increased use of the technique, there has been a growing incidence of obstetric complications in pregnancies resulting from IVF, raising concerns among patients, health professionals and researchers.

Placenta accreta is histologically defined as the complete or partial absence of the decidua basalis at the placental insertion site (Jauniaux *&* Ayres-de-Campos, 2018). Currently, PAS disorders are divided into three categories, as postulated by Luke *et al.* (1966) (Imafuku *et al.*, 2021): placenta accreta, when the villus only adheres to the myometrium; placenta increta, when the villus invades the myometrium; and placenta percreta, when the invasion includes the uterine serosa or adjacent organs. Prenatal diagnosis of this condition is essential for safe birth planning. A multidisciplinary approach in highly complex centers is essential to reduce morbidity and mortality in these cases (Jauniaux *&* Ayres-de-Campos, 2018). This obstetric complication has been increasingly associated with pregnancies resulting from assisted reproduction techniques, especially IVF. The relationship between IVF and placenta accreta has been the subject of intensive studies to understand the underlying mechanisms and mitigate the associated risks.

This review explores the complex intersection between in vitro fertilization and placenta accreta, examining the risk factors, pathophysiological mechanisms involved, and the most frequent complications. In addition, recent advances in understanding the pathogenesis of placenta accreta in pregnancies after IVF are presented, emphasizing strategies to minimize maternal and fetal complications. By elucidating the complex interactions between assisted reproductive technologies and adverse obstetric consequences, this article seeks to contribute to the advancement of scientific and clinical knowledge, promoting best practices in reproductive and gestational health care.

## MATERIALS AND METHODS

### Research Strategy

Systematic review with comprehensive research in the electronic databases PubMed, Cochrane Library, BVS, Embase, Web of Science, Scopus. The search did not limit the initial date of publication, with the final date being January 8, 2025. The search terms used are listed in Table 1.

**Table 1:** Search Terms.

Assisted Reproductive Technologies	Fertilization *in Vitro* [MeSH]
*In Vitro* Fertilization
Test-Tube Fertilization
Infertility treatment
Reproductive Techniques, Assisted [MeSH]
Assisted Reproductive
Assisted reproduct
Assisted conception
Embryo Transfer [MeSH]
Embryo Transfer
Intracytoplasmic Sperm Injection [MeSH]
Intracytoplasmic Sperm Injection
Spectrum of placenta accreta	Placenta Accreta [MeSH]
	Placenta Accreta
	Placenta Percreta
	Morbidly Adherent Placenta
	Placenta Accreta Spectrum
	Placenta increta
	Adherent placenta
	Placental disorders

### Inclusion and Exclusion Criteria

The inclusion criteria were: original studies published and fully avaiable in scientific databases or in printed versions; studies comparing an intervention group (pregnancy through IVF) to a control group (spontaneous pregnancy) in relation to the occurrence of placenta accreta; and original language English or Portuguese.

The exclusion criteria were: isolated case studies or case series with fewer than 10 patients; studies not fully available in the databases searched; articles not peer-reviewed, such as opinions, editorials and letters to the editor; review studies; studies that did not present specific data on IVF and placenta accreta; studies without a control arm; and articles written in languages other than English or Portuguese.

### Study Selection

**Figure 1:**
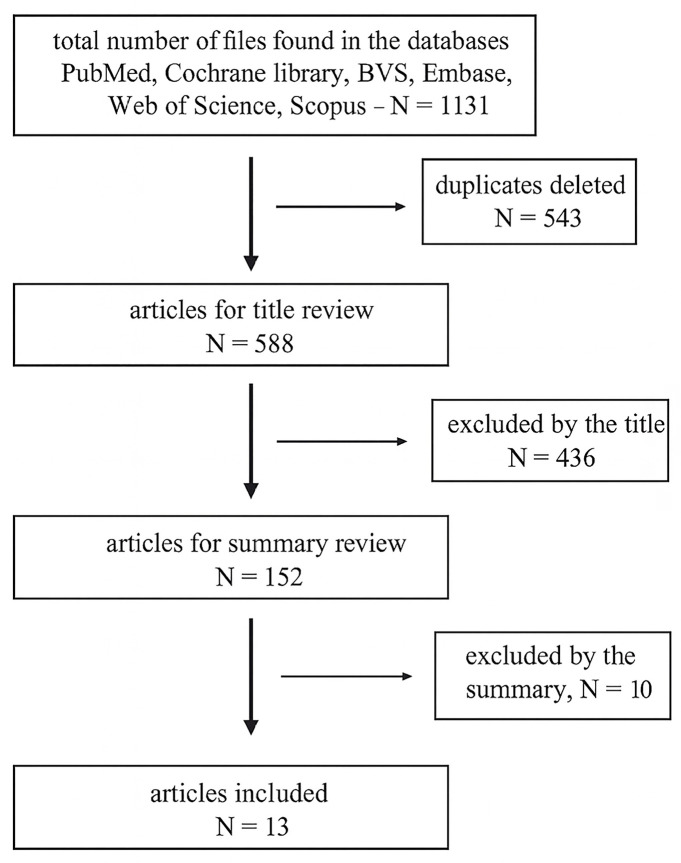
Study Selection.

Study selection was performed in three stages: identification, screening, and inclusion. In the identification stage, all titles and abstracts retrieved through the database search were reviewed by two independent reviewers, with the help of the Rayyan program. In the screening stage, the full texts of potentially relevant articles were assessed for inclusion and exclusion criteria. Disagreements between reviewers were resolved by consensus (Figure 1).

### Data Extraction

Data were extracted in a standardized manner using a Microsoft Excel extraction form. The extracted information included: author, year of publication, country of study, study design, sample size, demographic characteristics of participants (such as maternal age, previous obstetric history), definition of placenta accreta used, main results (expressed as relative risk (RR) for cohort studies and odds ratio for case-control studies) and authors’ conclusions. In addition, data were collected on potential confounders and statistical adjustments performed.

### Study Quality Assessment

The methodological quality of the included studies was assessed using the New Castle-Ottawa risk of bias assessment tool for cohort and case-control studies. Each study was classified as having low, moderate, or high risk of bias. Quality assessment was performed independently by two reviewers, with disagreements resolved by consensus.

### Data Analysis

The extracted data were qualitatively synthesized, with a detailed description of the main findings of each study.

### Ethical Considerations

Since this was a systematic literature review and did not involve direct patient involvement or primary data collection, it was not necessary to obtain approval from a research ethics committee. In line with good research practices, the analysis followed the PRISMA (Preferred Reporting Items for Systematic Reviews and Meta-Analyses) guidelines to ensure transparency and quality of the review.

## RESULTS

Thirteen articles were included in the review (Tables 2 and 3), comprising 252,295 pregnancies conceived by assisted reproductive technology (in vitro fertilization or intracytoplasmic sperm injection) and 16,834,591 spontaneous pregnancies, used as a control group. Few studies analyzed the difference in maternal outcomes between placenta accreta after ART and spontaneous conception.

**Table 2:** Comparison of the included studies.

Author	Year	Period	Country	Outline	N cases	IVF	Case- control	Age IVF	Age SC	Control (PP/CS)	RR PAS IVF (CI)	PAS Diagnosis	Maternal outcome
Zhu *et al*.	2016	2006-2014	China	RC	7923	2641	5282	31	31	CS	2.37 (1.9-2.95)	U	no
Tanaka *et al*.	2020	2015-2017	Japan	RC	6952	811	6141	37.2	31	X	7.35 (3.2-16.6)	U	no
Esh-Broder *et al*.	2011	2004-2009	Israel	Cohort	42	12	30	37.8	33.5	yes	13.2 (6.7-25.8)	P	no
Nagata *et al*.	2019	2011-2014	Japan	Cohort	103099	3147	90506	36	31	CS	6.85 (3.88-12.13)	C	yes
Modest *et al*.	2021	2013-2018	USA	RC	28344	1418	26926	36.2	32.1	yes	5.5 (3.4-8.7)	P	yes
Zhang *et al*.	2024	2018-2021	China	RC	47596	3203	41956	31.8	29.4	no	2.72 (2.42-3.07)	U	no
Wang *et al*.	2021	2009-2018	China	RC	10766	3836	6930	33.4	33.39	^*^	^*^	U	no
Violette *et al*.	2023	x	USA	RC	96480	48240	48240	35	33	yes	2.06 (1.44-2.93)	U	no
Lin *et al*.	2022	2014-2020	China	RC	14099	1489	12610	X	X	CS	^**^	U	no
Kong *et al*.	2022	2013-2018	China	RC	16535852	183059	16352793	X	X	no	2.0 (1.96-2.04)	U	no
Jiang *et a*l.	2021	2016-2017	China	RC	3212	1209	2003	31.8	30.4	no	2.12 (1.42-3.17)	P/C	no
Al-Khatib *et al*.	2023	2006-2020	USA	RC	240259	2651	237608	X	X	no	1.66-2.63	U	no
Imafuku *et al*.	2021	2010-2019	Japan	RC	4146	579	3566	X	X	no	4.1 (2.4-7.1)	P	no

Legend: CS = Cesarean Section; PP = Placenta Previa; SC = spontaneous conception; RC = retrospective cohort; U = unspecified; P = pathological; C = clinical; ART = assisted reproductive technology; FET = frozen embryo transfer.

**Table 3:** Newcastle-Ottawa classification of quality of non-randomized studies.

Author/year	Selection	Comparability	Outcome
Wang *et al*. (2021)	3	0	2
Violette *et al*. (2023)	3	2	3
Lin *et al*. (2022)	4	1	3
Kong *et al*. (2022)	3	1	2
Jiang *et al*. (2021)	3	2	2
Al-Khatib *et al*. (2024)	3	0	3
Zhu *et al*. (2016)	4	2	3
Nagata *et al*. (2019)	4	2	3
Zhang *et al*. (2024)	3	0	3
Tanaka *et al*. (2020)	3	1	1
Esh-Broder *et al*. (2011)	4	1	3
Modest *et al*. (2021)	3	2	2
Imafuku *et al*. (2021)	3	2	1

The articles included were published between 2011 and 2025. Most studies were conducted in China (46%), followed by the United States of America (23%) and Japan (23%). The studies were mostly retrospective cohort studies, with some being case-control or prospective cohort studies. The criteria for diagnosing PAS were anatomopathological in 30% of the studies, clinical in 15% and unspecified in 55%.

Zhu *et al.* (2016) reported an increased risk of PAS in both nulliparous and multiparous patients and a 20-fold increase in the risk of multiple pregnancies after IVF. He also observed that performing ICSI did not increase the risk for those patients who underwent IVF. In the retrospective cohort study conducted by Tanaka *et al.* (2020), the relative risk of PAS after ART was 7.35 (CI 3.2-16.6); however, 90% of the cases evaluated involved FET. Among the studies that address this association, Al-Khatib *et al.* (2024) stands out, which compared the prevalence of PAS after fresh-ET and FET, demonstrating that, although both correlate with a higher risk of PAS, when compared with spontaneous conception, pregnancies resulting from FET had a higher RR than those from fresh-ET. This data was also highlighted in the Imafuku *et al.* (2021) study.

The study conducted by Zhang *et al.* (2024) identified a relative risk of 2.72 (CI 2.42-3.07) for the development of placenta accreta after the use of assisted reproductive technologies. In this analysis, the relative risk was 1.46 (CI 1.19-1.79) for placenta accreta after fresh embryo transfer and 3.89 (CI 3.39-4.46) after frozen embryo transfer. In addition, the study found an association between endometrial thickness less than 9 mm and an increased risk of placenta accreta in patients undergoing frozen embryo transfer (Zhang *et al.*, 2024).

Lin *et al*. (2022) compared the incidence of PAS between IVF and spontaneous conceptions and conducted a specific analysis among women with a previous cesarean section. Even in this group, the study found that the risk of developing placenta accreta spectrum doubled if the method of conception was IVF. Furthermore, it was observed that 90% of patients with a previous cesarean section, regardless of whether they conceived by ART or spontaneously, had a new cesarean section as an outcome in the subsequent pregnancy (Lin *et al.*, 2022).

The study carried out by Nagata *et al.* (2019), was the first to evaluate maternal outcomes in patients with placenta accreta after assisted reproduction. The results showed a worse maternal outcome in pregnant women whose pregnancy was the result of assisted reproduction. A relative risk of 2.58 (CI 1.11-6.01) for intensive care unit(ICU) admission was observed in IVF patients and 3.76 (CI 1.68-7.06) for ICSI patients. The need for replacement of blood components after delivery was also higher in the IVF group, RR of 3.85 (CI 2.52-5.88), and ICSI, RR of 3.76 (CI 2.49 - 5.66). This analysis was adjusted for potential confounding factors, reinforcing the association between assisted reproduction and adverse outcomes in cases of placenta accreta (Nagata *et al.*, 2019).

In contrast, the cohort study conducted by Modest *et al.* (2021) reported that pregnant women diagnosed with placenta accreta after assisted reproduction treatments had lower rates of ICU admission compared to pregnant women with placenta accreta resulting from spontaneous conception. However, those diagnosed with placenta accreta after IVF had fewer diagnoses performed in the prenatal period, since they often did not present the risk factors traditionally associated with placenta accreta. In addition, patients undergoing IVF were less likely to develop placenta previa and had fewer previous cesarean sections compared to the control group, showing that assisted reproduction technology is an independent risk factor for the emergence of PAS (Modest *et al.*, 2021). The studies by Imafuku *et al.* (2021), as well as that by Kong *et al.* (2022), also corroborate this association as an independent risk factor.

In Jiang *et al.* (2021), it was observed that ART significantly increased the risk of developing gestational diabetes mellitus (GDM), RR of 1.42, premature rupture of membranes, RR of 1.65, and postpartum hemorrhage, RR of 1.38, in twin pregnancies, whose controls were other multiple pregnancies. Furthermore, an association was identified between IVF/ICSI and placenta accreta, with a relative risk of 2.12 (CI 1.42-3.17), which was also related to previous cesarean sections (Jiang *et al.*, 2021). The study conducted by Kong *et al.* (2022) also observed that pregnancies resulting from IVF were more likely to develop gestational complications, including gestational hypertension (RR 1.55 CI 1.51-1.59), preeclampsia (RR 1.54 CI 1.51-1.57), preterm birth (RR 1.48 CI 1.46-1.51), fetal distress (RR 1.39 CI 1.37-1.42) and fetal growth restriction (RR 1.36 CI 1.30-1.42).

The same study (Jiang *et al.*, 2021) revealed that 86.9% of monochorionic pregnancies were conceived spontaneously, while in dichorionic pregnancies 50% resulted from IVF and 50% occurred spontaneously. However, no significant association was found between assisted reproductive therapy and the occurrence of placenta previa, as in study by Esh-Broder *et al.* (2011). In contrast, the study carried out by Violette *et al.* (2023) identified an increased risk of placenta previa in pregnancies resulting from assisted reproductive therapy, with a relative risk of 2.98 (CI 2.64-3.35), and an even higher risk, of 11.3 (CI 5.86-21.8), for the occurrence of vasa previa, specifically in these pregnancies. The same study indicated that the relative risk of association between placenta previa and placenta accreta in the same patient after assisted reproduction therapy was 2.8 (CI 1.32-5.92) (Violette *et al.*, 2023).

Wang *et al.* (2021) analyzed the etiology of infertility in relation to the risk of placenta accreta and found significance only in patients with tubal factor, presenting a relative risk of 1.61 (CI 1.19-2.18), and with male factor, of 2.05 (CI 1.08-3.87). Endometriosis, ovulatory factor or the combination of multiple factors did not show a significant relationship with the development of placenta accreta (Wang *et al.*, 2021).

## DISCUSSION

The review of 13 articles and more than 17 million pregnancies reveals a significant gap in the analysis of specific maternal outcomes associated with placenta accreta after ART, compared with spontaneous pregnancies. The predominance of retrospective cohort studies, together with a variety of diagnostic criteria for PAS, emphasizes the complexity in directly comparing risks between the studied groups. The high representation of Chinese studies is notable, given the one-child policy that was in force for decades, influencing patient demographics and complicating the interpretation of results regarding the impact of previous uterine scars on post-ART pregnancies.

These results highlight the importance of rigorous and specialized monitoring during post-ART pregnancies, given the greater complexity and potential for complications, such as PAS. These are patients who require more rigorous prenatal monitoring, with ultrasounds for placental investigation. Studies (Birkmeyer *et al.*, 2002; Wright *et al.*, 2010) have shown that prenatal diagnosis of PAS, with birth in reference centers, reduces morbidity and mortality at birth, as well as the need for blood component replacement. Nieto-Calvache *et al.* (2022) found a better detection rate of accreta in prenatal tests in reference centers.

In a study conducted by Salmanian *et al.* (2020), pregnancies resulting from ART had an 8.7 times higher risk of PAS; however, in patients with one previous cesarean section, the RR was 10.1 (CI 5.1-20.2) and with four or more cesarean sections, the RR was 49.1 (CI 17-141.7). In this same study, it was observed that the diagnosis of placenta previa constitutes an RR of 94.6 (CI 29.3-305.1) for the occurrence of PAS. Among the 37,461 patients included, 5 had the three risk factors analyzed, with 100% of them developing PAS. This demonstrates that, although IVF is an independent risk factor for PAS, it presents a lower risk than the traditionally related risk factors, but it also adds significant risk when added to them (Salmanian *et al.*, 2020).

The exact pathophysiology of placenta accreta is not yet fully understood and remains a topic of ongoing investigation. However, several risk factors for its occurrence are well established, including previous cesarean section, placenta previa, advanced maternal age, multiparity, previous uterine curettage, and a history of uterine surgeries that resulted in scarring of the uterus (Esh-Broder *et al.*, 2011). In IVF, uterine embryo transfer occurs through the internal cervical canal using a catheter, which can induce uterine contractions and the release of prostaglandins. This process can alter the interaction between the embryo and the endometrium, impacting the placental implantation process (Jauniaux *et al.*, 2020).

One possible mechanism to explain the association between FET and placenta accreta is that serum estradiol (E2) levels may modulate the degree of trophoblast invasion and the extent of vascular remodeling during implantation. In both humans and nonhuman primates, supraphysiological E2 concentrations may be related to abnormal placentation and its sequelae, such as preeclampsia and fetal growth restriction. In FET cycles, E2 levels are typically lower than in fresh stimulation cycles, which may allow the window for trophoblast invasion to be prolonged. After exposure to high peak E2, the endometrium becomes refractory to implantation within 24 hours (Salmanian *et al.*, 2020).

The relationship between placenta accreta and maternal mortality is widely recognized and documented. As early as O’Brien *et al.* (1996) reported a 7% mortality rate associated with placenta percreta, although they emphasized that this figure probably underestimates the true risk. The main cause of maternal death associated with placenta accreta is massive hemorrhage, often accompanied by coagulation disorders (O’Brien *et al.*, 1996). It is noteworthy that maternal hemorrhage continues to be one of the main causes of maternal mortality worldwide today (PAHO - Pan American Health Organization & WHO -World Health Organization, 2017). These data reinforce the importance of early diagnosis of this condition during prenatal care, allowing detailed birth planning in tertiary care centers, with a multidisciplinary team prepared to deal with possible complications (American College of Obstetricians and Gynecologists & Society for Maternal-Fetal Medicine, 2018).

Betran *et al.* (2021), when analyzing global data between 1990 and 2018, identified that more than half of births in Brazil are the result of cesarean sections. Worldwide, approximately 1 in 5 children are born by cesarean section. Betran *et al.* (2021) projection is that this number will increase to 1 in 3 births by 2030. With the increasing number of cesarean sections and the increase in pregnancies through in vitro fertilization, the proportion of patients with multiple risk factors for placenta accreta spectrum will increase. In this context, it will be crucial to differentiate and identify specific cases to be screened during prenatal care, allowing for adequate planning, aiming for a gestational outcome with lower morbidity.

In summary, while ART offers a valuable opportunity for many couples to conceive, the challenges associated with post-ART pregnancy, particularly in relation to placenta accreta, require an integrated and multidisciplinary approach to optimize maternal and perinatal outcomes. Further research is essential to fully elucidate the underlying mechanisms and develop effective preventive strategies.

### Limitations

It is important to highlight that the study may be susceptible to the influence of confounding factors, since logistic regression was used to control for possible distorting variables. For example, patients undergoing IVF had a higher mean age than the control group, which in itself is already a known risk factor for several obstetric complications, including placenta accreta. In addition, the IVF group also showed a higher prevalence of other pathological conditions, such as metabolic disorders, which may contribute to an increased risk of complications, regardless of the reproductive method used. These factors, if not adequately controlled, may influence the results of the study and make it difficult to interpret a direct causal relationship between IVF and placenta accreta. Thus, although logistic regression attempts to adjust for these effects, the possibility of confounding bias still persists, requiring caution in generalizing the findings.
